# Improved Classification of Lung Cancer Using Radial Basis Function Neural Network with Affine Transforms of Voss Representation

**DOI:** 10.1371/journal.pone.0143542

**Published:** 2015-12-01

**Authors:** Emmanuel Adetiba, Oludayo O. Olugbara

**Affiliations:** ICT and Society Research Group, Durban University of Technology, P.O. Box 1334, Durban, 4000, South Africa; Harbin Medical University, CHINA

## Abstract

Lung cancer is one of the diseases responsible for a large number of cancer related death cases worldwide. The recommended standard for screening and early detection of lung cancer is the low dose computed tomography. However, many patients diagnosed die within one year, which makes it essential to find alternative approaches for screening and early detection of lung cancer. We present computational methods that can be implemented in a functional multi-genomic system for classification, screening and early detection of lung cancer victims. Samples of top ten biomarker genes previously reported to have the highest frequency of lung cancer mutations and sequences of normal biomarker genes were respectively collected from the COSMIC and NCBI databases to validate the computational methods. Experiments were performed based on the combinations of Z-curve and tetrahedron affine transforms, Histogram of Oriented Gradient (HOG), Multilayer perceptron and Gaussian Radial Basis Function (RBF) neural networks to obtain an appropriate combination of computational methods to achieve improved classification of lung cancer biomarker genes. Results show that a combination of affine transforms of Voss representation, HOG genomic features and Gaussian RBF neural network perceptibly improves classification accuracy, specificity and sensitivity of lung cancer biomarker genes as well as achieving low mean square error.

## Introduction

Lung cancer is a malignant tumor in the tissue of human lungs that remains one of the most leading causes of cancer related death cases worldwide [1]. Low dose Computed Tomography (CT) is the recommended standard for screening and early detection of lung cancer [2]. However, the survival rate of lung cancer is very low and more than half of patients diagnosed with the disease die within one year [3]. Lung cancer develops because of a sustained genetic damage to normal lung cells by carcinogens from cigarette smoke and other sources. More than 50 retrospective studies of smoking and lung cancer were reported to demonstrate a striking advancement in the risks of lung cancer for smokers or passive smokers compared to non-smokers [4]. In fact, recent studies [5,6] have attested to smoking as indisputably one of the leading causes of lung cancer, even though approximately 10% of lung cancer cases are attributed to the carcinogenic effects of radon gas, arsenic, nickel, asbestos, chromium and genetic susceptibility. The burning of tobacco in cigarette results in chemical processes such as pyrolysis, oxidation, hydrogenation, decarboxylation and dehydration of the constituents. Hence, over 3000 chemicals are produced out of which carcinogens responsible for cancers are phased into the particulate and vapor phases. The carcinogens in the particulate phase include benzo(a)pyrene, dibenz(a)anthracene, 5-methylchrysene, benzofluoranthenes, nicotine, N-nitrosonornicotene, catechol, nickel, cadmium and polonium. Similarly, the carcinogens in the vapor phase are hydrazine, vinyl chloride, urethane, formaldehyde, nitrogen oxides and nitrosodiethylamme. These gamuts of chemicals are either cancer initiators, complete carcinogens, tumor promoters or co-carcinogens. Consequently, they chemically activate the oncogenes and deactivate the tumor suppressor genes in the normal lung cell to produce mutations that result in tumors [7,8].

The availability of huge volumes of lung cancer mutation data has made the treatment of the disease fast advancing beyond the traditional approaches such as surgery, radiotherapy and chemotherapy. For a modern treatment of the disease, varieties of drugs to foster ‘personalized medicine’ have been developed to target the various genetic mutations towards stopping cancer growth before it becomes advanced and metastatic. These drugs have proven to be highly effective with fewer side effects in comparison with the traditional chemotherapies. Examples of targeted therapies approved for lung cancer treatment include gefitinib, erlotinib, bevacizumab, sorafenib and 28-amino-acid peptide (p28). These therapies target mutations in EGFR and TP53 [9–11]. However, the need to develop genomic based computational methods for classification, screening and early detection of lung cancer is highly decisive. This is because the recommended low dose CT is an imaging based technology that cannot be used for mutation detection [2,4,7,11]. Automatic genomic based classification, screening and early detection of lung cancer will go along way to help in recommending victims of known genetic mutations in the lung to take advantage of the available targeted therapies or participate in clinical trials for novel drugs.

In [12], DNA methylation markers and neural networks were reported as potentially viable tools for the automatic classification of lung cancer into Small Cell Lung Cancer (SCLC) and Non-Small Cell Lung Cancer (NSCLC). Markey et al. [13] developed a Classification And Regression Tree (CART) trained with 26 features to classify 41 clinical specimens as disease or non-disease. The features were computed from mass spectroscopy of blood serum samples of lung cancer and non-cancerous subjects using the mass-to-charge ratio and peak heights of proteins. Ramani and Jacob [14] designed a computational method using structural and physicochemical properties of protein sequences. They used the Bayesian network in their method to classify lung cancer tumors into SCLC, NSCLC and COMMON classes. Guan et al. [15] utilized Support Vector Machine (SVM), prior biological knowledge and Prediction Analysis for Microarray (PAM) to classify adenocarcinoma lung cancer. The aforementioned studies are necessary steps in the right direction, but unravelling the mutational contents of lung tumors has not been completely addressed in the literature. This implies that the promises of the targeted therapies to promptly arrest mutations in the lung may be elusive in the absence of relevant methods for screening and early detection of lung cancer mutations. Researchers have suggested that frequently mutated biomarker genes can be leveraged by designing kits for screening and early detection of lung cancer [16]. In line with this suggestion, a lung cancer prediction method was developed in [17]. The method was validated with data sets of EGFR, KRAS and TP53, which are the top three frequently mutated biomarker genes to predict mutations in lung cancer [16]. Ensemble and non-ensemble variants of Multilayer Perceptron (MLP) neural network and SVM were compared to predict six classes of biomarker genes and the best prediction accuracy of 95.90% was obtained using the MLP neural network ensemble [17].

The first overarching objective of this study is to extend the genomic coverage of the method reported in [17] to fourteen classes of the top ten frequently mutated lung cancer biomarker genes. It was emphasized in the literature that performance of classification algorithms can be affected for a large number of classes [18]. Consequently, the second objective of this study is to discover a set of affine invariant genomic features for improved classification of lung cancer biomarker genes despite the higher number of classes. This particular objective was achieved by exploring the Z-curve and tetrahedron affine transforms of Voss representation as well as the Histogram of Oriented Gradient (HOG). The Z-curve and tetrahedron affine transforms are used as nucleotides transformation methods because they intrinsically generate dimensionally reduced representation of Voss transformation with less computational cost [19,20]. Moreover, the affine transformed nucleotides are analogous to color image signals, which makes it easy to use the HOG method of the image processing domain to extract a set of genomic features for improved classification of lung cancer biomarker genes. The third objective of this study is to obtain an appropriate combination of computational methods for improved classification of lung cancer biomarker genes. Combinations of affine transforms of Voss representation, HOG method, MLP neural network and Gaussian Radial Basis Function (RBF) neural network we experimentally explored to achieve this objective.

## Materials and Methods

### Data Set

Normal (non-mutated) nucleotide sequences of ten different biomarker genes were obtained from the National Center for Biotechnological Information (NCBI) database. The reason for selecting the NCBI is that it is one of the most widely used databases in the Collaborative Consensus Coding Sequence (CCDS) consortium. The other CCDS databases are Ensembl Genome Browser, University of California Santa Cruz Genome Browser and Wellcome Trust Sanger Institute (WTSI) Genome Browser. The CCDS databases provide easy access to the same reference DNA sequence for any biomarker gene, regardless of the differences in the data and methods utilized for sequencing. The CCDS consortium tracks high quality identical protein annotations on the reference mouse and human genomes with a stable identification number named CCDS ID. The stability of the CCDS ID is because the consortium constantly makes efforts to ensure that existing CCDS are consistently updated by any collaborating member [21]. The symbol, description, CCDS ID and number of nucleotides of the top ten lung cancer biomarker genes used for this study are shown in Table 1.

**Table 1:** The characteristics of the top ten biomarker genes in lung cancer.

S/N	Gene Symbol	Gene Description	CCDS ID	Nucleotides
1	TP53	Tumor suppressor p53	CCDS 11118.1	1182
2	EGFR	Epidermal Growth Factor Receptor	CCDS 5514.1	3633
3	KRAS	Kirsten Rat Sarcoma viral oncogene homolog	CCDS 8702.1	567
4	KMT2C	Lysine (K)-specific Methyltransferase 2C	CCDS 5931.1	14736
5	CDKN2A	Cyclin-Dependent Kinase Inhibitor 2A	CCDS 6510.1	471
6	NF1	Neurofibromin 1	CCDS 11264.1	8457
7	STK11	Serine/Threonine Kinase 11	CCDS45896.1	1302
8	KMT2D	Lysine (K)-specific Methyltransferase 2D	CCDS44873.1	16614
9	ZNF521	Zinc Finger Protein 521	CCDS 32806.1	3936
10	SMARCA4	SWI/SNF related, Matrix associated, Actin dependent Regulator of Chromatin, subfamily A, member 4	CCDS12253.1	4944

Mutation data for this study were acquired from the Catalogue of Somatic Mutations in Cancer (COSMIC) database and they comprise of the top ten biomarker genes in lung cancer. The COSMIC database developed and hosted by the WTSI contains cases of curated and archived somatic mutations in the key cancer biomarker genes across many cancer samples [22]. The top ten biomarker genes in the COSMIC database with the highest frequency of mutations in the lung as at the time this study was carried out have symbols TP53, EGFR, KRAS, KMT2C, CDKN2A, NF1, STK11, KMT2D, ZNF521 and SMARCA4 [23]. The symbols were obtained from the HUGO Gene Nomenclature Committee (HGNC) database and most of these biomarker genes were specifically reported as frequently mutated biomarker genes in lung cancer [24–29]. In total, we extracted samples of 10784 lung cancer mutations and the data set utilized for our experimentation contains fourteen different classes, which are *Normal*, *EGFR Deletion*, *EGFR Substitution*, *KRAS Substitution*, *TP53 Deletion*, *TP53 Substitution*, *NF1 Substitution*, *KMT2C Substitution*, *CDKN2A Substitution*, *STK11 Deletion*, *STK11 Substitution*, *KMT2D Substitution*, *ZNF521 Substitution* and *SMARCA4 Substitution*.

The overall statistics of the curated and unique samples of normal and mutations data are shown in Table 2. The deletion mutation data for biomarker genes like KRAS, NF1, KMT2C, CDKN2A, KMT2D, ZNF521 and SMARCA in the COSMIC database are either non-existent or extremely few, which informed our decision to exclude them from our data samples.

**Table 2:** The statistics of the curated normal and mutation samples.

S/N	Description of Mutation	Count of Curated Samples	Unique Samples Curated for Experiment
1	Normal	100	100
2	TP53 Deletion	125	32
3	TP53 Substitution	1483	35
4	EGFR Deletion	1368	35
5	EGFR Substitution	2913	27
6	KRAS Substitution	4058	28
7	KMT2C Substitution	149	35
8	NF1 Substitution	88	35
9	CDKN2A Substitution	98	35
10	STK11 Deletion	32	32
11	STK11 Substitution	124	35
12	KMT2D Substitution	80	35
13	ZNF521 Substitution	105	35
14	SMARCA4 Substitution	61	35
	**TOTAL**	**10784**	**534**

### Transforming Genomic Nucleotides into Color Images

The gene as a basic unit of heredity is made up of a specific sequence of Deoxyribonucleic Acid (DNA) or Ribonucleic Acid (RNA). A DNA is a polymer made up of small molecules called nucleotides that can be distinguished by four bases. These bases are Adenine (A) = C_5_H_5_N_5_, Cytosine (C) = C_4_H_5_N_3_O, Guanine (G) = C_5_H_5_N_5_O and Thymine (T) = C_5_H_6_N_2_O_2_. Consequently, a DNA can be completely specified by a sequence consisting of the four alphabets {A, C, G, T}. The first essential step in the processing of a DNA sequence requires its conversion from a string of alphabets into the numerical equivalent [30–32]. Numerical characterization of DNA sequences can assist in contriving appropriate genomic features that capture the essence of the base composition and distribution in a quantitative manner. This could help in DNA sequence identification and comparison to detect the extent of genetic similarity or dissimilarity. The base composition provides the total content of each base in a DNA sequence and is easily determined. However, the base distribution, which is more difficult to determine is more informative and it gives a better discrimination amongst various genes even if the base composition numbers are the same [31]. Consequently, both base composition and distribution of a DNA sequence can be explored to numerically characterize genomic sequences.

The particular numerical encoding method used, determines how well the base composition and distribution of a DNA sequence is captured. Many numerical encoding methods have been reported in the literature with each having its strengths and weaknesses [33]. The Voss transformation is one of the most commonly used methods for numeric encoding of nucleotides [34,35]. It is an efficient spectral detector of the base distribution and periodicity features [33] and it represents DNA sequences with four binary indicator sequences as:

$$u_{b}[n]=\left\{ \begin{array}{l} 1,\;u_{b}[n]=b\\ 0,\;u_{b}[n]\neq b \end{array}\right.,\;\forall \;b\in \{A,\;C,\;G,\;T\},\;n=0,1,\ldots ,N-1$$ (1)

where 1 denotes the presence of the base b, at location n, 0 signifies its absence at that location and N is the length of the DNA sequence being encoded. However, the Voss representation is highly redundant [33]. Some other existing methods such as the Z-curve and tetrahedron affine transformations can be used to address the redundancy in the Voss representation [36]. The Z-curve and tetrahedron representations reduce the computational cost in the later processing stages of DNA sequences.

The Z-curve transformation was developed to encode DNA sequences with more biological semantics [37]. It uses a suitable geometrical representation to reduce the number of Voss representations from four to three in a compact way that is symmetric to all the four bases. The Z-curve contains all the information carried by the corresponding DNA sequences and therefore, the analysis of a DNA sequence can be performed by studying the corresponding Z-curve [20]. The 3-dimensional Z-curve vectors are expressed as [20,36]:

$$\begin{array}{l} x_{r}[n]=u_{A}[n]-u_{C}[n]+u_{G}[n]-u_{T}[n]\\ x_{g}[n]=u_{A}[n]+u_{C}[n]-u_{G}[n]-u_{T}[n],\;n=0,1,\ldots ,N-1\\ x_{b}[n]=u_{A}[n]-u_{C}[n]-u_{G}[n]+u_{T}[n] \end{array}$$ (2)

The tetrahedron transformation is similar to the Z-curve transformation, wherein the four nucleotide bases are transformed into 3-dimensional vectors that point from the center of a tetrahedron to its vertices. These 3-dimensional vectors are defined as [36–37]:

$$\begin{array}{l} x_{r}[n]=\frac{\sqrt{2}}{3}(2u_{T}[n]-u_{C}[n]-u_{G}[n])\\ x_{g}[n]=\frac{\sqrt{6}}{3}(u_{C}[n]-u_{G}[n]),\;n=0,1,\ldots ,N-1\\ x_{b}[n]=\frac{1}{3}(3u_{A}[n]-u_{C}[n]-u_{G}[n]-u_{T}[n]) \end{array}$$ (3)

where *r*, *g* and *b* in the subscript of the vectors are red, green and blue indicators. In fact, tetrahedron transformation has been referred to in the literature as the ‘rgb’ transformation of a DNA sequence [33].

In order to efficiently process the rgb vectors (Eqs 2 and 3) to obtain the corresponding rgb images, an appropriate number of windows that corresponds to the image height (H), an appropriate window size that corresponds to the image width (W) and the overlap are chosen to define three HxW dimensional matrices. In this study, the number of windows was determined based on the DNA sequence length (N) in the biomarker gene. The window size of 200 and an overlap of 50 nucleotides were used [38,39]. The matrices were normalized within the range of 0–255 to portray each of them as a grayscale image. These three grayscale images are rendered as a color image in the rgb color space.

### Pattern Classification and Feature Extraction

The task of pattern classification to be performed by a pattern classifier essentially involves the cataloguing of raw data into desired classes based on the intrinsic patterns in the data. Automatic pattern classification has been accurately performed in various application areas using machines [40]. The complexity of a pattern classifier heavily depends on the dimension of the feature vector and the number of the training data samples. A compact or low dimensional feature representation that retains the descriptive contents of the original data set is highly desirable for efficient memory requirement, speeding up processing time and minimizing computational complexity of a pattern classifier. Some of the existing feature extraction and dimensionality reduction methods in statistics are Factor Analysis (FA), Independent Component Analysis (ICA) and Principal Component Analysis (PCA).

In signal and image processing domain, several other methods have been developed to extract representative features of an original data set that result in dimension reduction. These methods include Vector Quantization (VQ), Scale Invariant Feature Transform (SIFT), Speeded Up Robust Features (SURF), Principal Component Analysis SIFT (PCA-SIFT), Local Binary Patterns (LBP) and Histogram of Oriented Gradient (HOG) [41–44]. The HOG is particularly described in the literature as a strong shape, appearance and texture extraction method [43–45]. We have selected HOG method for use in this study because of its attractive properties such as better invariance to illumination. Moreover, an earlier study has shown that the HOG method outperformed the LBP method for the extraction of compact genomic features [17]. In the original implementation of the HOG method, a 3x3 block of cells and 9 bins were used to generate a feature vector of 81 elements from a grayscale image and tested to be ideal for pedestrian detection [44]. However, because of the low dimensions of some genomic images, we applied minimum dimensions of 2x2 block of cells and 9 bins to generate a compact HOG genomic feature vector of 36 elements from a grayscale image. The grayscale image was obtained from a color image of DNA sequence using the MATLAB. The extracted HOG genomic features were subsequently fed into a pattern classifier to classify lung cancer biomarker genes.

In this study, two rival state-of-the-art pattern classifiers explored for the classification of lung cancer biomarker genes are the Multilayer Perceptron (MLP) neural network and Radial Basis Function (RBF) neural network. They are widely used to solve the problems of pattern classification and function approximation [46–58]. However, the pattern classifiers have intrinsic strengths and weaknesses because of their distinguishing properties. MLP neural networks have the capability to implicitly detect complex non-linear associations between independent and dependent variables. However, they require greater computational resources and are prone to the problem of overfitting. On the other hand, RBF neural networks have a strong advantage of being simple to design, they have a good generalization capability, they perform robustly and are tolerant of input noise [59]. Nevertheless, they may not perform better than MLP neural networks in all circumstances. The performance of each pattern classifier will obviously depend on the nature of the problem being considered. MLP neural networks may produce a more fitted output to cross validation data set than RBF neural networks, but RBF neural networks require less trials and error than MLP neural networks. In addition, each pattern classifier may perform differently for diverse approximation functions. Since the underlying function that approximates our experimental data was unknown beforehand, we found it prudent to experiment with the two pattern classifiers to discover the one that performs well for the classification task in this study.

### Experimental Models and Performance Evaluation

**Fig 1:**
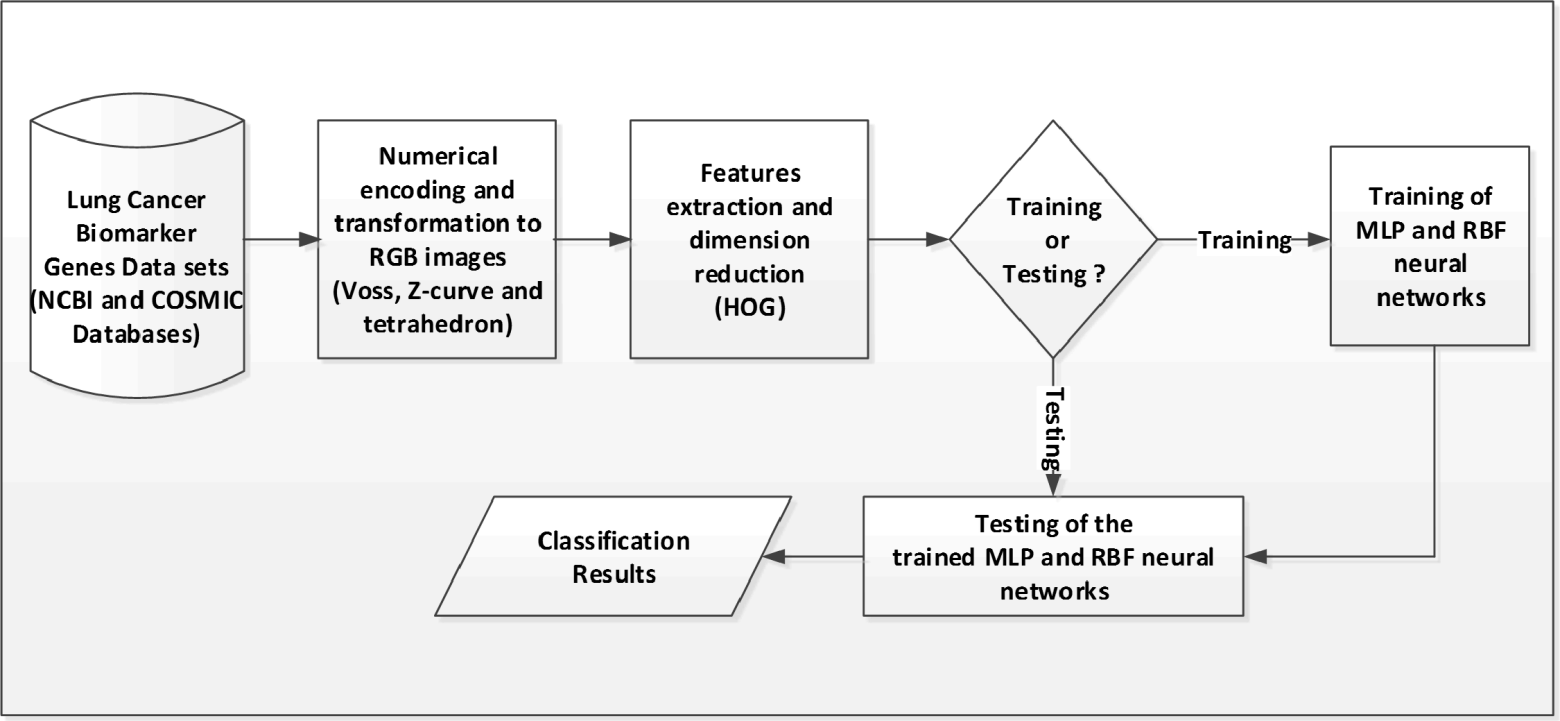
The generic architecture of the experimental models.

Four experimental models were considered in this study to discover a set of affine invariant genomic features and to determine an appropriate combination of computational methods for improved classification of lung cancer biomarker genes. Fig 1 shows the design of a generic architecture for the four experimental models. The experimental models were implemented using the MATLAB R2012a programming environment. Based on the experimental models, experiments were performed on a computer that contains an Intel Core i5-3210M CPU, which operates at 2.50GHz speed, 6.00GB RAM, 500 GB Hard disk and runs 64-bit Windows 8 operating system. In all the four experimental models, the data set was partitioned into 70% training, 15% testing and 15% validation. In the first experimental model, the Z-curve representation was used to obtain a color image from the Voss representation, HOG method was used to generate a genomic feature vector of 36 elements from the color image and MLP neural network was used to classify the feature vector. In the second experimental model, the tetrahedron representation was used instead of the Z-curve representation used in the first experimental model. Consequently, changing the encoding method from the Z-curve to the tetrahedron is the difference between the first and the second experimental models. In the third experimental model, the Z-curve representation was used to obtain a color image from the Voss representation, HOG method was used to generate a genomic feature vector of 36 elements from the color image and Gaussian RBF neural network was used to classify the feature vector. The fourth experimental model was designed to use the tetrahedron representation instead of the Z-curve representation, which is the only difference between this fourth experimental model and the third experimental model.

The configurations of the MLP neural networks for the first and the second experimental models are the same. There are 36 neurons in the input layer because the HOG genomic feature vector has 36 elements. The output layer of the MLP neural network contains 14 neurons because there are 14 classes in the genomic data set. It has been suggested that more hidden layers with a high number of neurons usually lead to fewer local minima [60]. Hence, two hidden layers were considered and the neural network was tested with 100, 200, 300, 400 and 500 neurons to experimentally determine the appropriate number of neurons for each of the hidden layers. The MLP neural network uses a linear activation function in the input layer to transmit the exact features without any transformation. The hyperbolic tangent function was used in the neurons in the hidden and output layers to fully take advantage of their nonlinearity and differentiability properties. These properties are essential qualities for optimal performance of MLP neural networks [60]. Moreover, the MLP neural network was configured with 500 training epochs, learning rate of 0.1, maximum training time of 120sec, minimum performance gradient of 1e-6, validation checks of 500 and performance goal of 0.

The configurations of the Gaussian RBF neural networks in the third and fourth experimental models are the same. The Gaussian RBF neural networks were configured to have the MSE goal of 0, spread of 0.1, 36 neurons in the input layer and 14 neurons in the output layer. These configurations are based on the number of elements in each feature vector and the number of biomarker gene classes in the data set. However, a Gaussian RBF neural network normally contains one hidden layer and automatically adds neurons to the hidden layer until it meets the specified mean squared error goal. The training of the Gaussian RBF neural networks was stopped when the number of hidden layer neurons reached the maximum default value of 534, which is the number of instances in the training data set.

Four different performance metrics commonly used in the literature to assess the performance of a pattern classifier were used to quantitatively evaluate the performances of MLP and Gaussian RBF neural network pattern classifiers. These performance metrics are the accuracy, Mean Square Error (MSE), specificity and sensitivity. The accuracy of a pattern classifier can be computed from the confusion matrix as the percentage of correctly classified entities. This is equivalent to the sum of diagonal elements of the confusion matrix divided by the total number of elements in the classes. The MSE is the mean of the square of the difference between the expected output and the actual output of a pattern classifier. The probability that a pattern classifier correctly classifies a non-positive instance, as negative is called specificity or True Negative Rate (TNR). The probability that a pattern classifier labels the instances of the target class correctly is called sensitivity or True Positive Rate (TPR). The Receiver Operating Characteristics (ROC) is the plot of sensitivity against 1-specificity to graphically illustrate the relationship between sensitivity and specificity of a pattern classifier [60–62].

## Experimental Results

**Fig 2:**
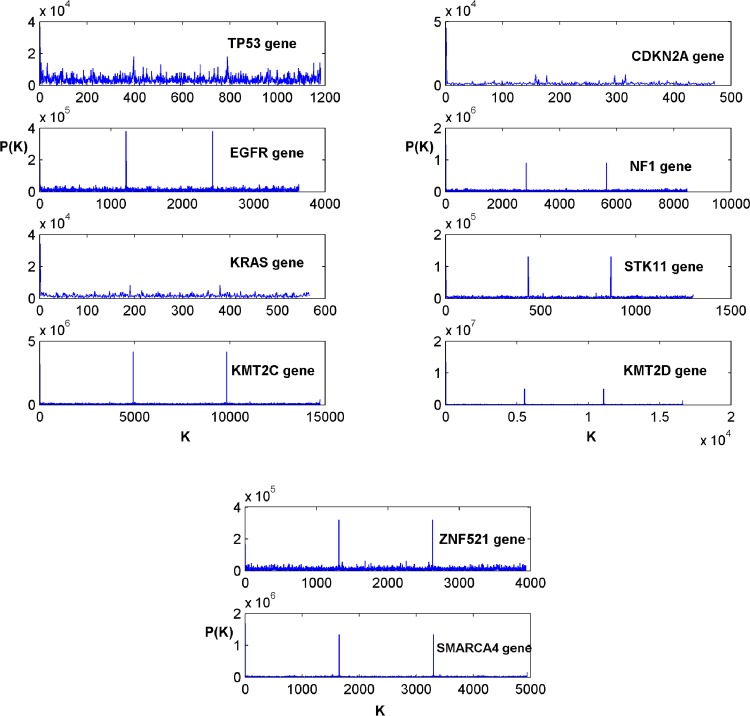
The Z-curve power spectral of the biomarker genes in Table 1.

**Fig 3:**
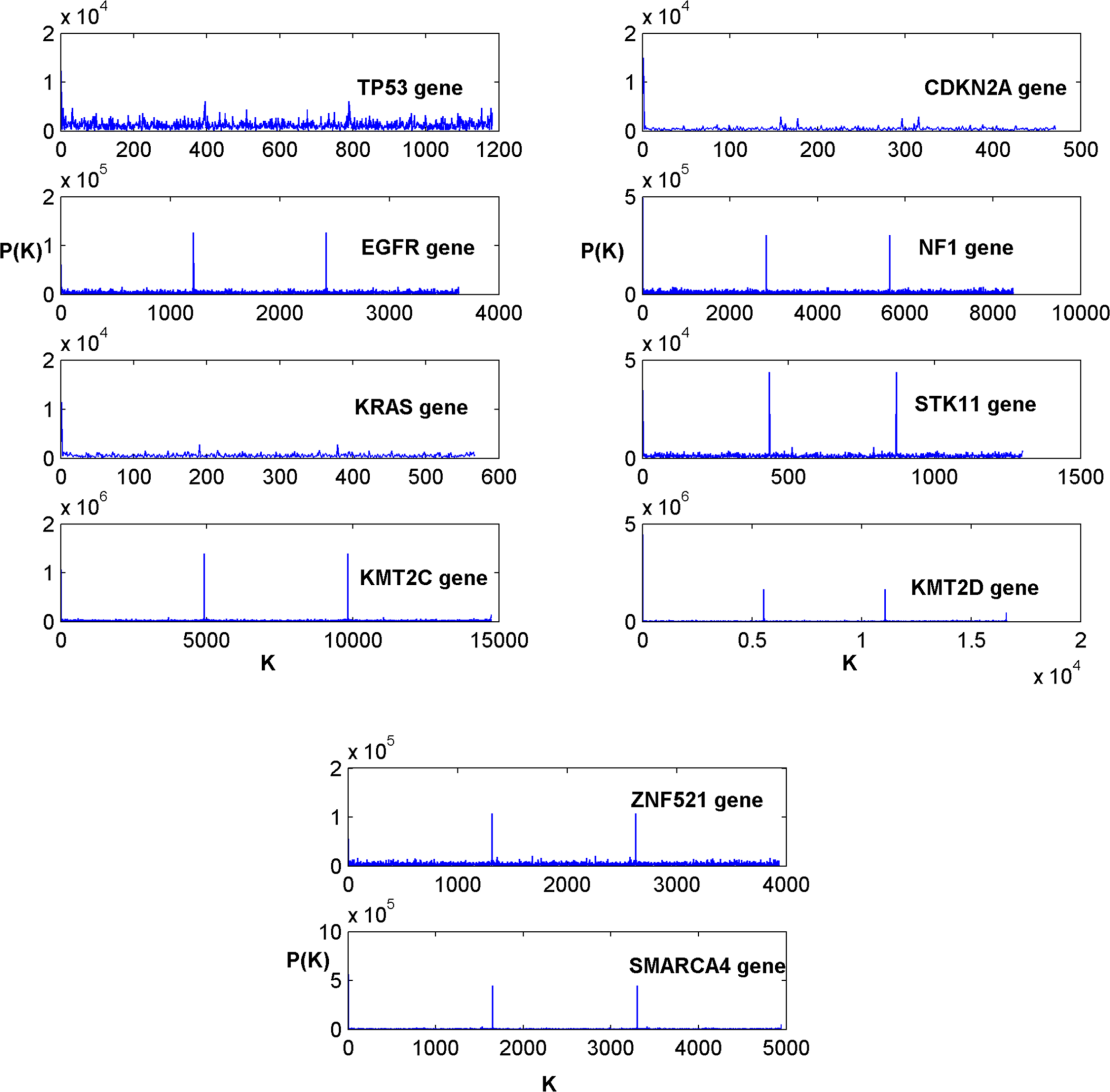
The tetrahedron power spectral of the biomarker genes in Table 1.

The comparative results of the Z-curve and tetrahedron transforms are first presented to ascertain whether the feature sets obtained with respect to the two affine transforms are invariant. Figs 2 and 3 respectively show the power spectrum plots of the Z-curve and tetrahedron representations of DNA sequences of biomarker genes in Table 1. Each corresponding spectrum shape obtained using the Z-curve representation (Fig 2) can be seen to be highly similar to that obtained using the tetrahedron representation (Fig 3). This result gives an indication of a strong similarity between the Z-curve and tetrahedron representations. The Z-curve spectral shapes of the biomarker genes are uniquely different from each other (Fig 2) and the same trend is observed across the shapes of the biomarker genes obtained using the tetrahedron representation (Fig 3). It can be observed from the two figures, that the spectral shapes of the TP53 biomarker gene have dense spectral details with spectral envelopes of high amplitudes. Conversely, the spectral shapes of the EGFR biomarker gene in the two figures contain dense spectral details of low amplitudes with two spikes of high amplitudes at K = 1200 and K = 2400. The spectral shapes of the KRAS biomarker gene in both figures have thin spectral details that terminate before K = 600 without showing any conspicuous spike. The spectral shapes of the KMT2C biomarker gene have flat spectral details with high amplitude spikes at K = 5000 and K = 10000 in both figures. Similar to the spectral shapes of the KRAS biomarker gene, the spectral shapes of the CDKN2A biomarker gene in both figures have thin spectral details that terminate before K = 500 unlike the spectral shapes of the KRAS biomarker gene that terminate after K = 500. The spectral shapes of NF1, STK11, KMT2D, ZNF621 and SMARCA4 biomarker genes all have two spikes of different amplitudes at different values of K, which is an indication of the uniqueness of these biomarker genes.

**Fig 4:**
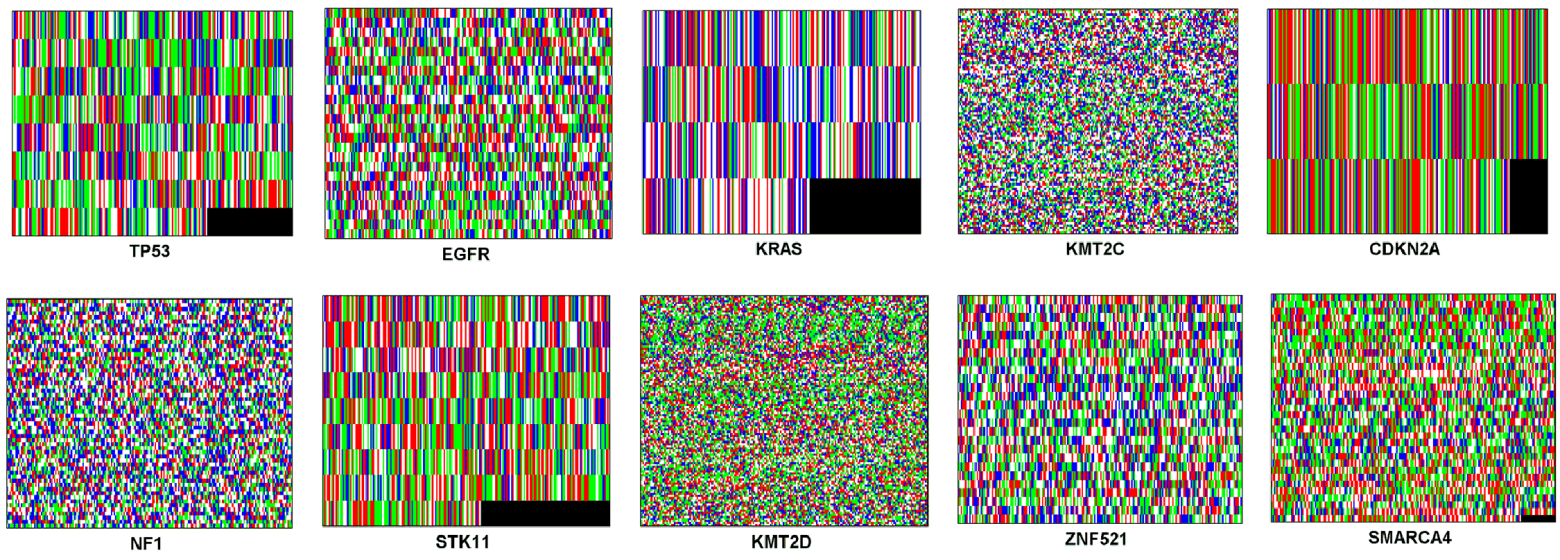
The Z-curve transformed color images of all the biomarker genes in Table 1.

**Fig 5:**
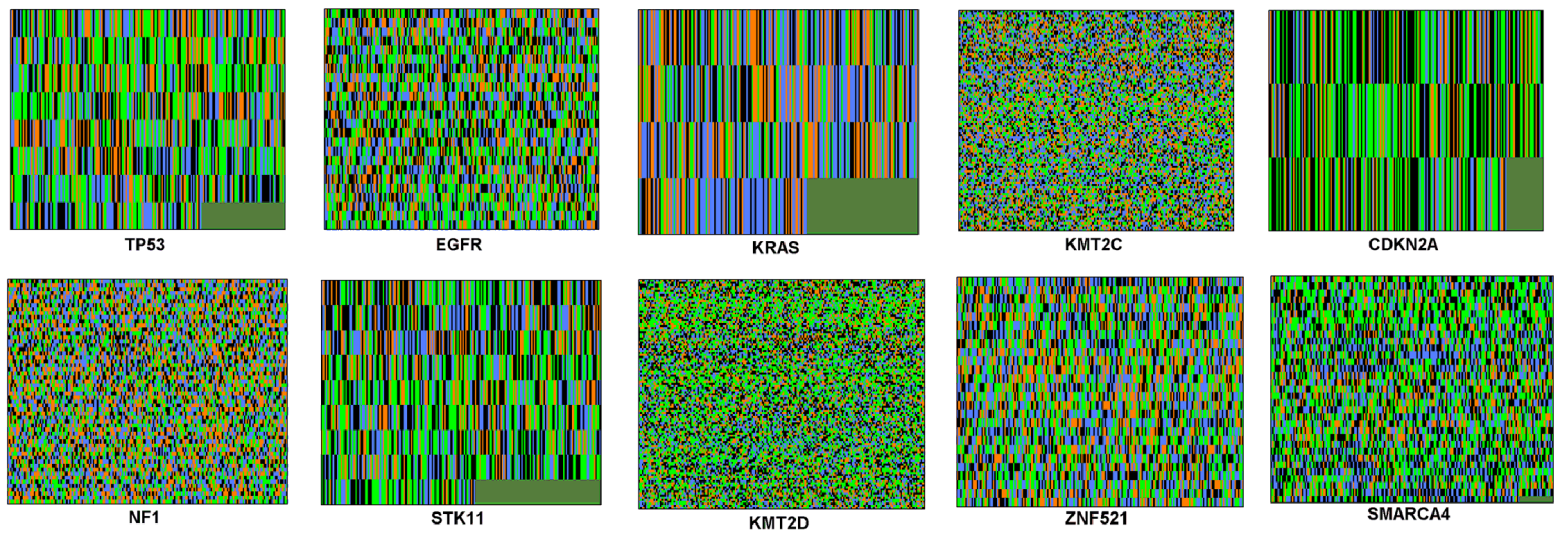
The tetrahedron transformed color images of all the biomarker genes in Table 1.

In addition, the color images obtained using the Z-curve and tetrahedron representations of all the biomarker genes in Table 1 are respectively shown in Figs 4 and 5. It is clearly observed through the subjective visual inspection that the textures of the corresponding images of biomarker genes obtained using the two affine transforms are similar. Moreover, it can be seen that the images of TP53, KRAS, CDKN2A and STK11 biomarker genes have heavy textures and contain conspicuous black or green patches at the bottom right corner of the images. The textures of the images of EGFR, ZNF521 and SMARCA4 in both figures are coarse with only the image of SMARCA4 having very tiny black or green patch at the bottom right corner. However, the images of KMT2C, NF1 and KMT2D biomarker genes have soft textures. Even though the textures of the corresponding images are similar across each biomarker gene, their colors are different.

An objective evaluation by quantitatively analyzing the image textures was performed to complement the results of subjective evaluation of image textures of the biomarker genes (Figs 4 and 5). In doing this, we computed the Haralick second order statistical values of contrast and homogeneity [63]. High contrast values are usually expected for heavy textures and low values for soft textures. Homogeneity values are the inverse of contrast values and the higher the contrast, the lower the homogeneity and vice versal. The Haralick values obtained for each of the color images of the ten biomarker genes obtained using the Z-curve and tetrahedron representations are shown in Table 3. The table shows that the contrast values of the Z-curve transformed color images rank in a similar manner as those of the tetrahedron transformed color images (value in bracket denotes the rank of a biomarker gene). For the Z-curve transformed color images, the KRAS biomarker gene ranks first with the highest contrast value of 13099 while KMT2D biomarker gene ranks last with a contrast value of 6358. Meanwhile, for the tetrahedron transformed color images, the CDKN2A biomarker gene ranks first with the highest contrast value of 13495 while KMT2D biomarker gene ranks last with a contrast value of 6392.

**Table 3:** Contrast and homogeneity statistical textures for color images of the normal biomarker genes.

Symbol of a Biomarker Gene	Z-Curve Transformed Image		Tetrahedron Transformed Image	
	Contrast	Homogeneity	Contrast	Homogeneity
KRAS	**13099 (1)**	**0.0342 (1)**	**12997 (2)**	**0.0347 (3)**
CDKN2A	**12474 (2)**	0.0345 (2)	**13495 (1)**	0.0342 (2)
TP53	12414 (3)	**0.0346 (3)**	12512 (3)	**0.0339 (1)**
STK11	12364 (4)	0.0368 (4)	12177 (4)	0.0376 (4)
EGFR	11006 (5)	0.0391(7)	11042 (5)	0.0382 (7)
ZNF521	10975 (6)	0.0383 (5)	11021 (6)	0.0386 (5)
SMARCA4	10450 (7)	0.0390 (6)	10509 (7)	0.0387 (6)
NF1	8801 (8)	0.0421 (8)	8815 (8)	0.0417 (8)
KMT2C	6779 (9)	0.0438 (9)	6761 (9)	0.0436 (9)
KMT2D	6358 (10)	0.0445 (10)	6392 (10)	0.0448 (10)

The homogeneity values of the Z-curve transformed color images also rank in a similar manner as those of the tetrahedron transformed color images. For the Z-curve transformed color images, the KRAS biomarker gene ranks first with a homogeneity value of 0.0342 while KMT2D biomarker gene ranks last with a homogeneity value of 0.0445. However, for the tetrahedron transformed color images, the TP53 biomarker gene ranks first with a homogeneity value of 0.0339 while the KMT2D biomarker gene ranks last with a homogeneity value of 0.0448. Table 3 shows that only two out of the ten biomarker genes (KRAS and CDKN2A) have dissimilar rankings between the Z-curve and tetrahedron transformed color images with respect to the contrast values. In addition, the table shows that with respect to the homogeneity values, only two out of the ten biomarker genes (KRAS and TP53) have a dissimilar ranking between the Z-curve and tetrahedron transformed color images. This result implies that 20% of the biomarker genes are disimilarly ranked using the Z-curve and tetrahedron affine transforms. The differences in the ranking values of the contrast and homogeneity are as a result of the varying colors used by the two affine transforms to render the images of the biomarker genes.

In addition, a paired t-test was performed to determine if the differences between the sets of values of the contrast and homogeneity of the Z-curve and tetrahedron transformed color images are statistically significant. The mean difference between the two sets of the contrast values (M = 100.1, SD = 2.4e+03, N = 10) is significantly greater than zero, *h* = 0, *tsat* = -0.0912 and two-tail *p* = 0.9283. This statistic indicates a high probability of the result occurring under the null hypothesis of no difference between the mean of the two sets of contrast values. The null hypothesis is accepted, because *p*>0.05 (*p* = 0.9283) and a 95% CI about mean difference is (-0.0024, 0.0022). Similarly, the mean difference between the two sets of homogeneity values (M = 9e-5, SD = 0.0038, N = 10) is greater than zero, *h* = 0, *tstat* = 0.0529 and two-tail *p* = 0.9584. This statistic indicates a high probability of the result occurring under the null hypothesis of no difference between the means. The null hypothesis is accepted, because *p*>0.05 (*p* = 0.9584) and a 95% CI about mean difference is (-0.0035, 0.0037). The final result of the t-test further validates the invariance of the sets of genomic features obtained from the color images of the Z-curve and tetrahedron transformed DNA sequences.

The next stage concerns the results of the experiments performed based on the four experimental models of this study. In the first experiment, out of the five different experimental trials with varying number of hidden layer neurons of 100, 200, 300, 400 and 500, the MLP neural network configuration with 400 neurons in the hidden layer gave the best classification accuracy of 75.84%. The confusion matrix from where the accuracy value of this MLP neural network is computed is shown in Table 4. It can be seen in Table 4 that 90 out of the 100 instances are wrongly classified in the normal class of the dataset, which is not a good result. It can be observed in the table that 10 instances of the normal class were wrongly classified as TP53 deletion, EGFR substitution, KMT2C substitution, NF1 substitution, CDKN2A substitution, STK11 substitution, KMT2D substitution, ZNF521 substitution and SMARCA4 substitution respectively. Moreover, 2 instances of EGFR deletion were wrongly classified as normal, 5 instances of EGFR substitution were wrongly classified as KRAS substitution, 10 instances of KRAS substitution were wrongly classified as EGFR substitution, 10 instances of STK11 deletion were wrongly classified as STK11 substitution and 8 instances of STK11 substitution were wrongly classified as STK11 deletion.

**Table 4:** The confusion matrix of the computational method based on the Z-curve, HOG and MLP neural network.

a	b	c	d	e	f	g	h	i	j	k	l	m	n	Pattern classified as:
10	0	0	**2**	0	0	0	0	**4**	0	0	0	0	0	**a** = *Normal*
0	35	0	0	0	0	0	0	0	0	0	0	0	0	**b** = *TP53 Deletion*
**10**	0	27	0	0	0	0	0	0	0	0	0	0	0	**c** = *TP53 Substitution*
0	0	0	26	0	0	0	0	0	0	0	0	0	0	**d** = *EGFR Deletion*
**10**	0	0	0	27	**10**	0	0	0	0	0	0	0	0	**e** = *EGFR Substitution*
0	0	0	0	**5**	25	0	0	0	0	0	0	0	0	**f** = *KRAS Substitution*
**10**	0	0	0	0	0	35	0	0	0	0	0	0	0	**g** = *KMT2C Substitution*
**10**	0	0	0	0	0	0	35	0	0	0	0	0	0	**h** = *NF1 Substitution*
**10**	0	0	0	0	0	0	0	31	0	0	0	0	0	**i** = *CDKN2A Substitution*
0	0	0	0	0	0	0	0	0	22	**8**	0	0	0	**j** = *STK11 Deletion*
**10**	0	0	0	0	0	0	0	0	**10**	27	0	0	0	**k** = *STK11 Substitution*
**10**	0	0	0	0	0	0	0	0	0	0	35	0	0	**l** = *KMT2D Substitution*
**10**	0	0	0	0	0	0	0	0	0	0	0	35	0	**m** = *ZNF521 Substitution*
**10**	0	0	0	0	0	0	0	0	0	0	0	0	35	**n** = *SMARCA4 Substitution*

**Fig 6:**
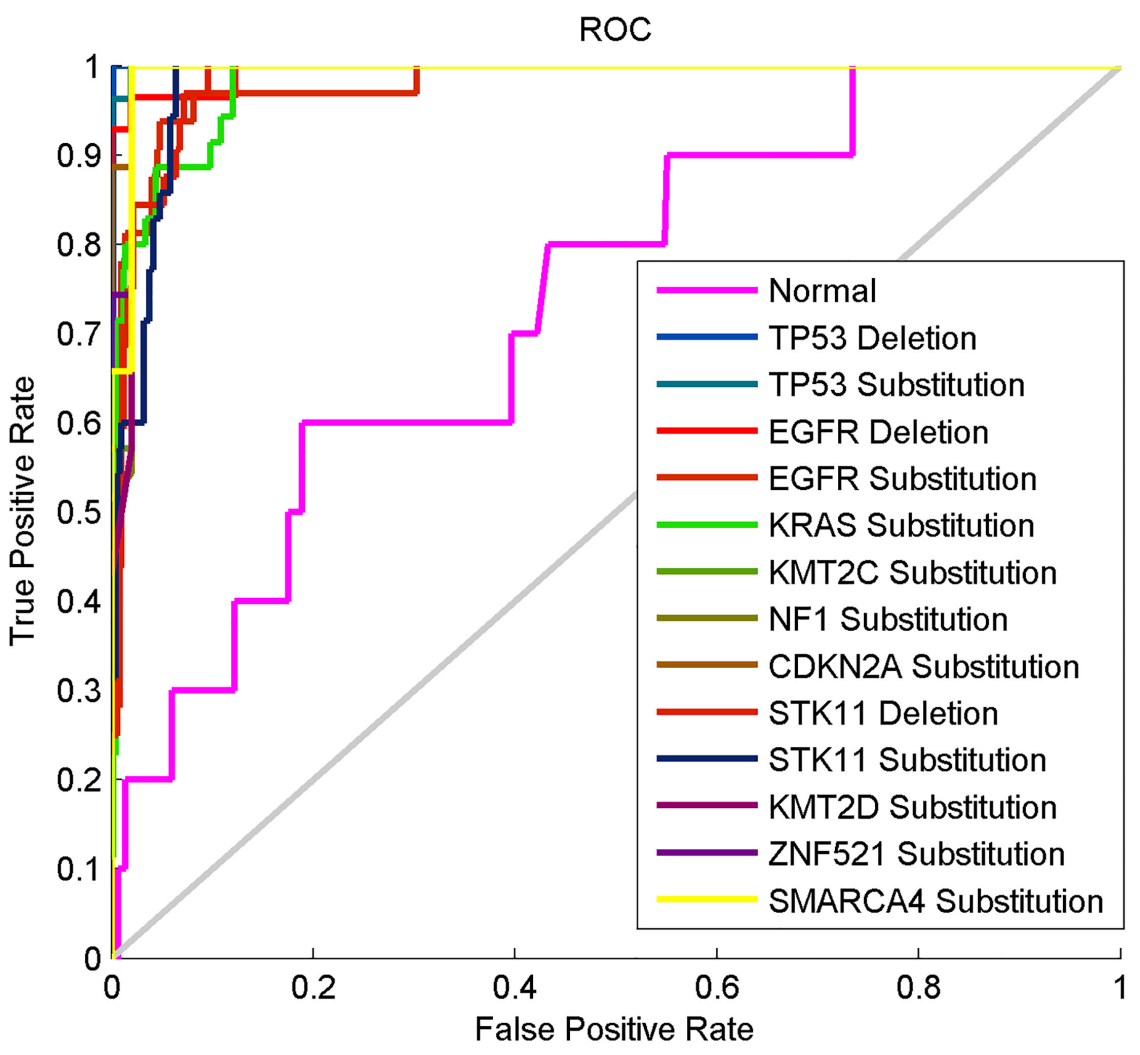
The ROC curves of the computational method based on the Z-curve, HOG and MLP neural network.

The ROC curves of the MLP neural network in the first experiment are shown in Fig 6. The sensitivity of 0.7654 and specificity of 0.9820 results were obtained from the ROC curves using appropriate functions in MATLAB R2012a. The ROC curve for perfect classification accuracy is usually the line connecting (0,0) to (0,1) and (0,1) to (1,1) [64]. As shown on the ROC curves in Fig 6, the performance of the MLP neural network for the n*ormal* class is not at all close to perfect classification while its performance for the other 13 classes are better. None of the other 13 classes can however be said to achieve perfect classification accuracy with this current MLP neural network configuration as illustrated on the ROC curves, which are only slightly close to the perfect classification line.

In the second experiment, five trials of the MLP neural network with varying number of hidden layer neurons of 100, 200, 300, 400 and 500 were also tested. Overall, the MLP neural network configuration with 200 neurons gave the best classification accuracy of 73.97%. The confusion matrix for the MLP neural network configuration in the second experiment is shown in Table 5. The reported accuracy of 73.97% was computed from the confusion matrix as previously done in the first experiment. Table 5 shows that all the 100 instances of the normal class were wrongly classified. In particular, it can be seen that in other classes, 1 instance of the TP53 deletion was wrongly classified as TP53 substitution, 12 instances of EGFR substitution were wrongly classified as KRAS substitution, 5 instances of KRAS substitution were wrongly classified as EGFR substitution, 1 instance of STK11 deletion was wrongly classified as TP53 deletion while 20 instances of *STK11 deletion* were wrongly classified as STK11 substitution. There is no considerable improvement in the result of this second experiment when compared to the result of the first experiment.

**Table 5:** The confusion matrix of the computational method based on the tetrahedron, HOG and MLP neural network.

a	b	c	d	e	f	g	h	i	j	k	l	m	n	Pattern classified as:
0	0	0	0	0	0	0	0	0	0	0	0	0	0	**a** = *Normal*
0	34	0	0	0	0	0	0	0	**1**	0	0	0	0	**b** = *TP53 Deletion*
**10**	**1**	27	0	0	0	0	0	0	0	0	0	0	0	**c** = *TP53 Substitution*
**10**	0	0	28	0	0	0	0	0	0	0	0	0	0	**d** = *EGFR Deletion*
0	0	0	0	20	**5**	0	0	0	0	0	0	0	0	**e** = *EGFR Substitution*
**10**	0	0	0	**12**	30	0	0	0	0	0	0	0	0	**f** = *KRAS Substitution*
**10**	0	0	0	0	0	35	0	0	0	0	0	0	0	**g** = *KMT2C Substitution*
**10**	0	0	0	0	0	0	35	0	0	0	0	0	0	**h** = *NF1 Substitution*
**10**	0	0	0	0	0	0	0	35	0	0	0	0	0	**i** = *CDKN2A Substitution*
0	0	0	0	0	0	0	0	0	11	0	0	0	0	**j** = *STK11 Deletion*
**10**	0	0	0	0	0	0	0	0	**20**	35	0	0	0	**k** = *STK11 Substitution*
**10**	0	0	0	0	0	0	0	0	0	0	35	0	0	**l** = *KMT2D Substitution*
**10**	0	0	0	0	0	0	0	0	0	0	0	35	0	**m** = *ZNF521 Substitution*
**10**	0	0	0	0	0	0	0	0	0	0	0	0	35	**n** = *SMARCA4 Substitution*

**Fig 7:**
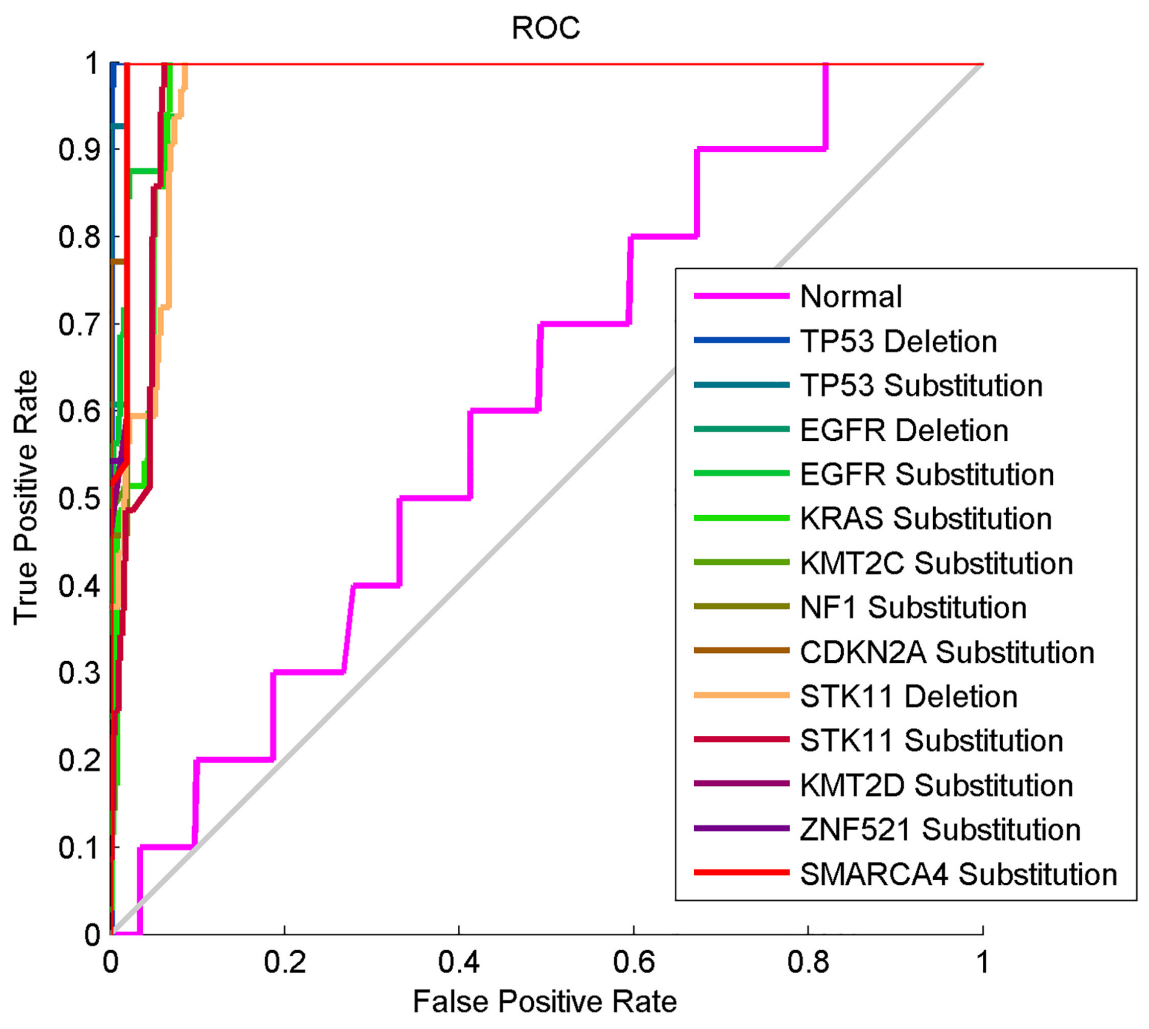
The ROC curves of the computational method based on the Z-curve, HOG and MLP neural network.

Fig 7 shows the ROC curves used to compute the sensitivity of 0.7143 and specificity of 0.9812. The ROC curves show that the normal class is poorly classified by the MLP neural network in this second experiment. Although the neural network performed better on the remaining 13 classes similar to what was obtained in the first experiment, the performance of the MLP neural network for the 13 classes is still not perfect. This is because the ROC curve for each of the 13 classes is just slightly close to the perfect classification line.

In the third experiment, the classification accuracy obtained from the Gaussian RBF neural network is 98.88%. The confusion matrix from which the accuracy was computed as was done in the first and second experiments is shown in Table 6. In this table, it can be seen that all the 100 instances of the normal class were correctly classified. However, 1 instance of NF1 substitution was wrongly classified as normal and 5 instances of KMT2D substitution were wrongly classified as normal. This result of the third experiment is a radical improvement when compared to the results of the first and second experiments.

**Table 6:** The confusion matrix of the computational method based on the Z-curve, HOG and Gaussian RBF neural network.

a	b	c	d	e	f	g	h	i	j	k	l	m	n	Pattern classified as:
100	0	0	0	0	0	0	**1**	0	0	0	**5**	0	0	**a** = *Normal*
0	35	0	0	0	0	0	0	0	0	0	0	0	0	**b** = *TP53 Deletion*
0	0	27	0	0	0	0	0	0	0	0	0	0	0	**c** = *TP53 Substitution*
0	0	0	28	0	0	0	0	0	0	0	0	0	0	**d** = *EGFR Deletion*
0	0	0	0	32	0	0	0	0	0	0	0	0	0	**e** = *EGFR Substitution*
0	0	0	0	0	35	0	0	0	0	0	0	0	0	**f** = *KRAS Substitution*
0	0	0	0	0	0	35	0	0	0	0	0	0	0	**g** = *KMT2C Substitution*
0	0	0	0	0	0	0	34	0	0	0	0	0	0	**h** = *NF1 Substitution*
0	0	0	0	0	0	0	0	35	0	0	0	0	0	**i** = *CDKN2A Substitution*
0	0	0	0	0	0	0	0	0	32	0	0	0	0	**j** = *STK11 Deletion*
0	0	0	0	0	0	0	0	0	0	35	0	0	0	**k** = *STK11 Substitution*
0	0	0	0	0	0	0	0	0	0	0	30	0	0	**l** = *KMT2D Substitution*
0	0	0	0	0	0	0	0	0	0	0	0	35	0	**m** = *ZNF521 Substitution*
0	0	0	0	0	0	0	0	0	0	0	0	0	35	**n** = *SMARCA4 Substitution*

**Fig 8:**
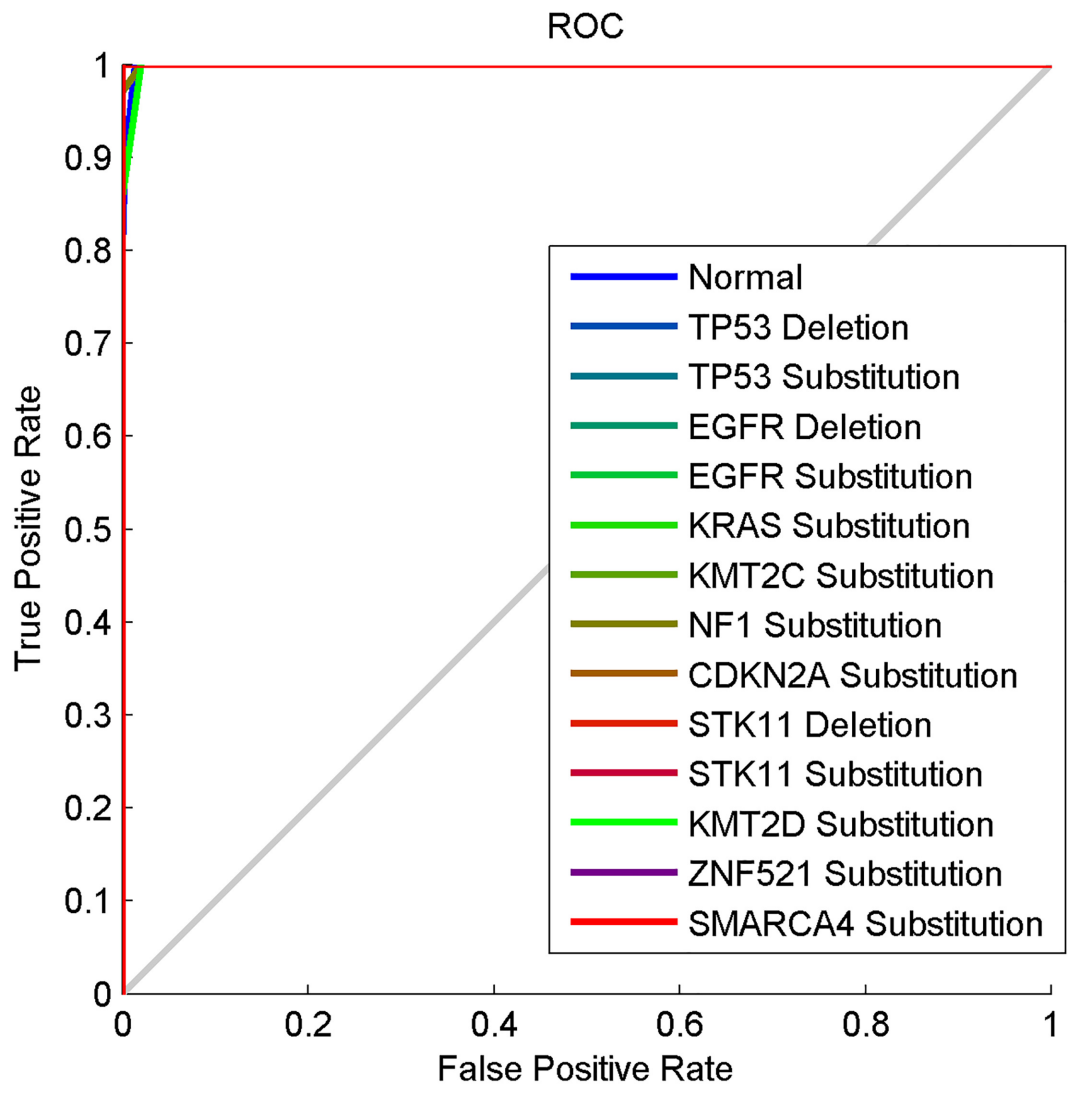
The ROC curve of the computational method based on the Z-curve, HOG and Gaussian RBF neural network.

Fig 8 shows the ROC curves used to calculate the sensitivity of 0.9960 and specificity of 0.9991. The curves clearly show that all the 14 classes were properly classified by the Gaussian RBF neural network in this third experiment. This is because all the curves overlap one another and are lined very closely or directly on the perfect classification line.

In the fourth experiment, the Gaussian RBF neural network gave classification accuracy of 98.50%. The confusion matrix of the neural network in this fourth experiment from which the reported accuracy was computed is shown in Table 7. It can be seen from the table that all the 100 instances in the normal class were also correctly classified. However, 1 instance of the KMT2C substitution was wrongly classified as normal, 1 instance of the STK11 substitution was wrongly classified as normal, 5 instances of KMT2D substitution were wrongly classified as normal and 1 instance of SMARCA4 substitution was wrongly classified as normal. This result of the fourth experiment is comparable to the result of the third experiment, but not better because 8 instances were wrongly classified when compared to 6 instances that were wrongly classified in the result of the third experimental model.

**Table 7:** The confusion matrix of the computational method based on the tetrahedron, HOG and Gaussian RBF neural network.

a	b	c	d	e	f	g	h	i	j	k	l	m	n	Pattern classified as:
100	0	0	0	0	0	**1**	0	0	0	**1**	**5**	0	**1**	**a** = N*ormal*
0	35	0	0	0	0	0	0	0	0	0	0	0	0	**b** = *TP53 Deletion*
0	0	27	0	0	0	0	0	0	0	0	0	0	0	**c** = *TP53 Substitution*
0	0	0	28	0	0	0	0	0	0	0	0	0	0	**d** = *EGFR Deletion*
0	0	0	0	32	0	0	0	0	0	0	0	0	0	**e** = *EGFR Substitution*
0	0	0	0	0	35	0	0	0	0	0	0	0	0	**f** = *KRAS Substitution*
0	0	0	0	0	0	34	0	0	0	0	0	0	0	**g** = *KMT2C Substitution*
0	0	0	0	0	0	0	35	0	0	0	0	0	0	**h** = *NF1 Substitution*
0	0	0	0	0	0	0	0	35	0	0	0	0	0	**i** = *CDKN2A Substitution*
0	0	0	0	0	0	0	0	0	32	0	0	0	0	**j** = *STK11 Deletion*
0	0	0	0	0	0	0	0	0	0	34	0	0	0	**k** = *STK11 Substitution*
0	0	0	0	0	0	0	0	0	0	0	30	0	0	**l** = *KMT2D Substitution*
0	0	0	0	0	0	0	0	0	0	0	0	35	0	**m** = *ZNF521 Substitution*
0	0	0	0	0	0	0	0	0	0	0	0	0	34	**n** = *SMARCA4 Substitution*

**Fig 9:**
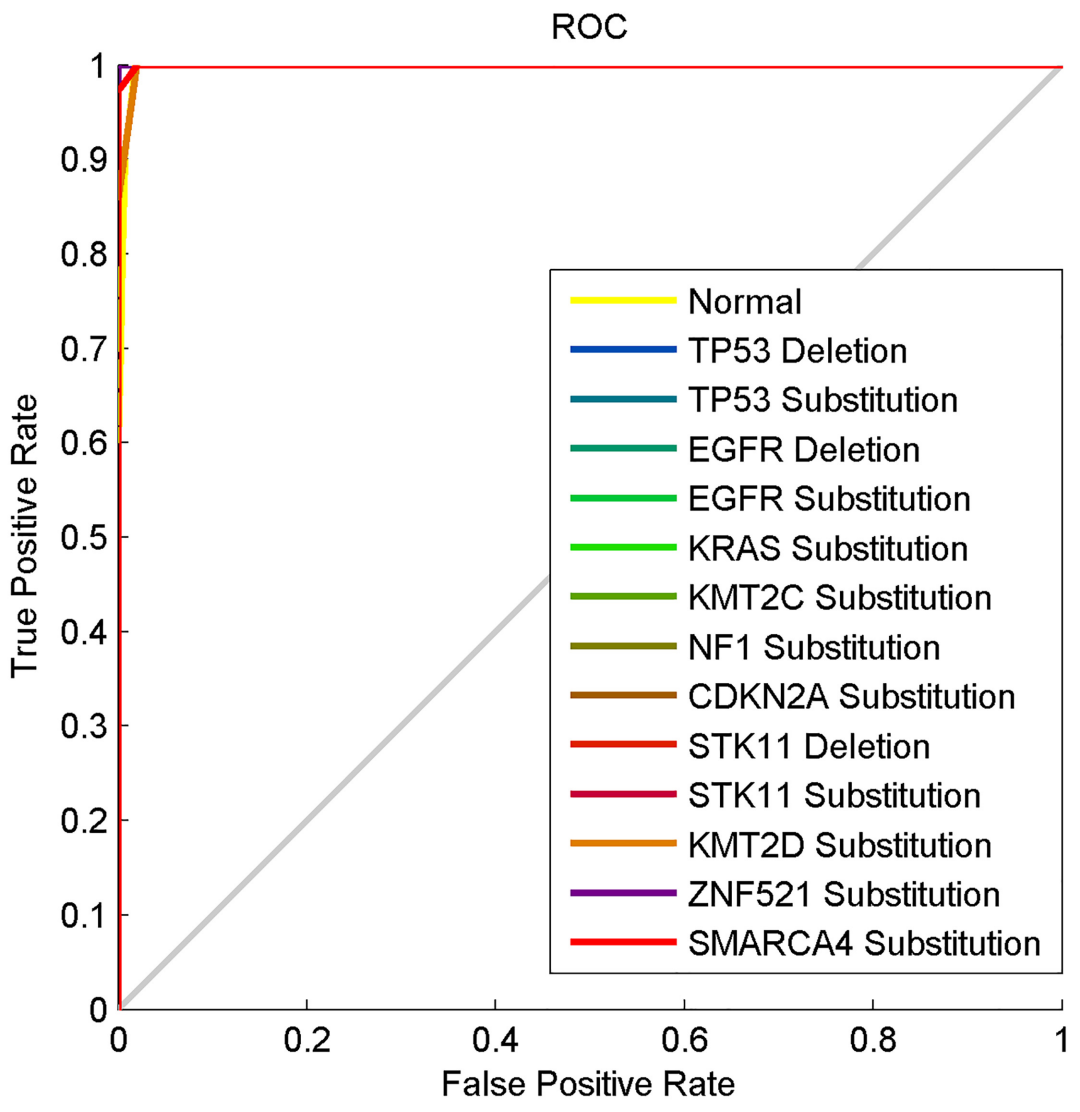
The ROC curve of the computational method based on the tetrahedron, HOG and Gaussian RBF neural network.

Fig 9 shows the ROC curves of all the 14 classes used to calculate the sensitivity of 0.9947 and specificity of 0.9989. The curves show that all the 14 classes were well classified in the fourth experiment because all the curves closely align or fall directly on the perfect classification line. These ROC curves are very close to the curves we obtained in the third experiment, which further corroborate the closeness of the results obtained from the third and fourth experiments.

The final result obtained from all the four experimental models in this study is summarized in Table 8. The MSE values were computed for the MLP and Gaussian RBF neural network pattern classifiers and reported in Table 8. It can be seen in this table that the results obtained using a combination of computation methods based on affine transforms of Voss representation, HOG and Gaussian RBF neural network (that is experimental models 3 and 4) are better than the results obtained using a combination of affine transforms of Voss representation, HOG and MLP neural network (that is experimental models 1 and 2). This final result shows that Gaussian RBF neural network performs better than MLP neural network for the classification task considered in this study.

**Table 8:** The accuracy, MSE, specificity and sensitivity obtained from the four different experimental models of this study.

Experimental model	Affine Transformation	Pattern Classifier	Accuracy	MSE	Specificity	Sensitivity
1	Z-curve	MLP	0.7584	0.0240	0.9820	0.7654
2	Tetrahedron	MLP	0.7397	0.0273	0.9812	0.7143
3	Z-curve	RBF	0.9888	0.0011	0.9991	0.9960
4	Tetrahedron	RBF	0.9850	0.0016	0.9989	0.9947

## Discussion

The results of the power spectral analysis as shown in Figs 2 and 3 were used in this study to establish the invariance of the affine transforms of the Voss representation of lung cancer biomarker genes. The spectrum of each biomarker gene has a unique shape for the Z-curve affine transformation (Fig 2). This trend can also be observed in the spectrum of the tetrahedron affine transformation of each biomarker gene (Fig 3). In agreement with this result, the similarity of the two affine transforms was established by Shao et al. [36]. The authors showed that there is a strong similarity between the Signal to Noise Ratio (SNR) curves obtained from the Z-curve and tetrahedron representations of the Homo sapiens mitochondrion DNA sequences [36].

The sets of HOG genomic features obtained from the color images of the affine transformed biomarker genes were also established to be invariant in this study. This invariance was established by calculating the paired t-test of the Haralick contrast and homogeneity values obtained from the color images (Table 3). The paired t-test results indicate no statistically significant difference between the two sets of HOG genomic features obtained from the color images of the affine transformed biomarker genes. The results of the four experimental models of this study (Table 8) also established the invariance of the two sets of HOG genomic features used for improved classification of the lung cancer biomarker genes.

Each experimental model is unique with respect to the combined methods and variation in the results obtained based on the accuracy, MSE, specificity and sensitivity. The first and second experimental models utilized the same configuration of MLP neural network and HOG genomic features. However, despite the similarity in the pattern classifiers and feature extraction methods, higher accuracy, specificity, sensitivity and lower MSE were obtained with the first experimental model compared to the second experimental model. It can be deduced that an improved classification was recorded using the first experimental model because of the influence of the Z-curve transformation. Although, using the first experimental model, we obtained a better classification result when compared to the second experimental model. The sensitivity of 0.7654 shows that the first experimental model did not result into classifying the instances of the normal class acceptably. This result is also observed in the confusion matrix of Table 4 in which 90 out of the 100 instances of the normal class were wrongly classified. However, the specificity of 0.9820 obtained with the first experimental model is an indication of a good performance on mutated instances of our data set. This is also observed in Table 4, in which merely 39 out of the 434 mutated instances were wrongly classified. Meanwhile, because of the leaning of the result towards the mutated data set only, we cannot recommend a system based on the first experimental model as being good enough for the improved classification of the lung cancer biomarker genes.

The third experimental model as shown in Table 8 has led to obtain better classification result than the first and second experimental models across all the evaluation metrics. The third experimental model differs from the first experimental model with respect to the pattern classifier employed. In addition, the third experimental model differs from the second experimental model with respect to the transformation method and pattern classifier employed. The accuracy of 98.88%, MSE of 0.011, specificity of 0.9991 and sensitivity of 0.9960 were obtained with the third experimental model. These performance values indicate that a system based on the third experimental model is able to classify both the normal and the mutated instances of the dataset adequately and acceptably. This assertion is also supported by the result in the confusion matrix shown in Table 6, in which all the 100 instances of the normal class were correctly classified and 428 of the 434 mutated instances were correctly classified. The immediate inference we can draw from this radical improvement in the result obtained using the third experimental model when compared to the results obtained using the first and the second experimental models is the use of Gaussian RBF neural network. This inference is further corroborated by the fact that the result obtained from the fourth experimental model is comparable to the result obtained from the third experimental model. Similar to the third experimental model, the fourth experimental model has led to improved classification of both normal and mutated instances of the dataset adequately and acceptably. This is illustrated with the accuracy of 98.5%, MSE of 0.0016, specificity of 0.9989 and sensitivity of 0.9947. This result is further espoused by the result shown in the confusion matrix (Table 7), in which all the 100 instances of the normal class were correctly classified and 426 of the 434 mutated instances were correctly classified.

The little difference in the results obtained in the third and fourth experimental models may be attributed to the different colors used in rendering the Z-curve and tetrahedron transformed images of the biomarker genes. This slight difference can also be seen in Table 3 as conveyed by the Haralick contrast and homogeneity values. Largely, the results obtained from the third and the fourth experimental models can be regarded as improvements over the results reported in [17]. The three most frequently mutated genes in lung cancer used in [17] were also the first three genes in this current study (Table 1). In that earlier study [17], despite the fewer number of biomarker genes used, the combination of the Voss representation, the HOG genomic features and the MLP ensemble gave the classification accuracy of 95.90% and MSE of 0.0159. Both third and fourth experimental models of this current study are improvements of the result in [17] despite the increase in the number of biomarker genes from three to ten coupled with the use of a single Gaussian RBF pattern classifier rather than a more complicated ensemble pattern classifier. Moreover, the accuracy of 98.88% obtained in this study is also an improvement over the accuracy of 87.6% obtained in a relatively similar study in [14]. These authors used Bayesian network and features from protein sequences of lung cancer tumors to classify the tumors into three classes of Small Cell Lung Cancer (SCLC), Non-Small Cell Lung Cancer (NSCLC) and COMMON [14]. The study objective of extending the genomic coverage of the method reported in [17] to the top ten biomarker genes in the COSMIC database [23] has been successfully achieved.

Based on the results obtained in the current study as displayed in Table 8, we recommend systems based on the Z-curve and tetrahedron affine transforms, HOG genomic features and Gaussian RBF neural network for improved classification of lung cancer biomarker genes. Thus far, we have been able to realize the three important objectives of this study. These objectives are to extend the genomic coverage of the architecture proposed in [17] from three to ten biomarker genes. To discover a set of affine invariant genomic features for improved classification of lung cancer biomarker genes despite the higher number of classes. To obtain the most appropriate combination of computational methods to achieve improved classification of lung cancer biomarker genes.

## Conclusion

The application of methods from the image processing domain to transform DNA sequences into the corresponding color images and the classification of lung cancer biomarker genes based on the genomic features extracted from the color images are the distinctive contributions of this study. We have successfully extended the genomic coverage of lung cancer classification to the top ten frequently mutated lung cancer biomarker genes with fourteen different classes. We have also found a set of affine invariant genomic features using the Z-curve, tetrahedron and Histogram of Oriented Gradient (HOG). We have performed experiments based on combinations of the Z-curve and tetrahedron affine transforms of Voss representation, Histogram of Oriented Gradient (HOG), Multilayer Perceptron (MLP) neural network and Gaussian Radial Basis Function (RBF) neural network. This was to experimentally obtain an appropriate computational methods for improved classification of lung cancer biomarker genes. Results show that a combination of affine transforms of Voss representation, HOG genomic features and Gaussian RBF neural network perceptibly improves the classification accuracy, specificity and sensitivity of lung cancer biomarker genes as well as achieving low mean square errors. This finding was validated with samples of top ten biomarker genes previously reported to have the highest frequency of lung cancer mutations and sequences of normal biomarker genes from the COSMIC and NCBI databases respectively.

The computational methods based on affine transforms of Voss representation, HOG genomic features and Gaussian RBF neural network as reported in this study can be readily implemented in a multi-genomic system for classification, screening, early detection and qualification of lung carcinoma victims for targeted molecular therapies. Another prospect of the computational methods is that their software implementation can be easily interfaced with the Next Generation Sequencing (NGS) platforms to detect lung cancer mutation profiles of at risk persons and those with an early onset of lung cancer. This will definitely help with recommending patients for targeted molecular therapies and ultimately reduce lung cancer mortality. Future work on the proposed computational methods will involve the incorporation of more biomarker genes and other genetic defects in the lung such as methylation, copy number alteration and loss of heterozygosity. In addition, we hope to explore other feature extraction methods and perform more intensive comparative studies on other state-of-the art machine learning methods to further enhance the performance of the computational methods.

## References

[1] Juergens RA, Brahmer JR (2006) Adjuvant treatment in non-small cell lung cancer: Where are we now? Natl Compr. Canc. Netw. 4: 595–600. 10.6004/jnccn.2006.0049 16813727

[2] Marshall HM, Bowman RV, Vang IA, Fong KM, Berg CD (2013) Screening for Lung Cancer with low-dose computed tomograph: a review of current status. Journal of thoracic disease, 5 (Suppl 5), S524–S539. 10.3978/j.issn.2072-1439.2013.09.06 24163745 PMC3804881

[3] Erik R, Andrew L Interpreting Health Benefits and Risks: A Practical Guide to Facilitate Doctor-Patient Communication Springer Cham Heidelberg, New York Dordrecht London, 10.1007/978-3-319-11544-3_1

[4] Garfinkel L (1981) Time trends in lung cancer mortality among nonsmokers and a note on passive smoking. J. Natl. Cancer Inst. 66: 1061–1066. 6941041 10.1093/jnci/66.6.1061

[5] Samet JM, Avila-Tang E, Boffetta P, Hannan LM, Olivo-Marston S, Thun MJ, et al (2009) Lung cancer in never smokers: clinical epidemiology and environmental risk factors. Clinical Cancer Res. 15:5626–5645. 19755391 10.1158/1078-0432.CCR-09-0376 PMC3170525

[6] Mehan MR, Ayers D, Thirstrup D, Xiong W, Ostroff RM (2012) Protein Signature of Lung Cancer Tissues. PLoS ONE 7(4): e35157 10.1371/journal.pone.0035157 22509397 PMC3324437

[7] Lawrence AL, Virginia LE, Kenneth EW, John A, John L (1984) Smoking and Lung Cancer: An Overview. Cancer Research 44: 5940–5958. 6388830

[8] Lin Q, Peng Q, Yao F, Pan XF, Xiong LW, Wang Y, et al (2012) A Classification Method Based on Principal Components of SELDI Spectra to Diagnose of Lung Adenocarcinoma. PLoS ONE 7(3): e34457 10.1371/journal.pone.0034457 22461913 PMC3312904

[9] Ravi S, Lung Cancer Research: From Prevention to Cure, Thoracic Oncology Research Program, Department of Medicine, Section of Hematology/Oncology, University of Chicago Cancer Research Center, Available: http://www.uchospitals.edu/pdf/uch_012779.pdf. Accessed 11 August 2015.

[10] Razzak M (2013) Targeted therapies: one step closer to drugging p53. Nature Reviews Clinical Oncology 10(5): 246–246. 10.1038/nrclinonc.2013.43 23507742

[11] Cornfield J, Haenszel W, Hammond EC, Lilienfeld AM, Shimkin MB, Wynder EL (2009) Smoking and lung cancer: recent evidence and a discussion of some questions. International journal of epidemiology 38(5): 1175–1191. 10.1093/ije/dyp289 19773415

[12] Marchevsky AM, Tsou JA, Laird-Offringa IA (2004) Classification of individual lung cancer cell lines based on DNA methylation markers: use of linear discriminant analysis and artificial neural networks. J. Mol. Diagn. 6:28–36. 14736824 10.1016/S1525-1578(10)60488-6 PMC1867460

[13] Markey MK, Tourassi GD, Floyed CE Jr (2003) Decision tree classification proteins identified by mass spectrometry of blood serum samples from people with and without lung cancer, Proteomics 3: 1678–1679. 12973724 10.1002/pmic.200300521

[14] Ramani RG, Jacob SG (2013) Improved Classification of Lung Cancer Tumors Based on Structural and Physicochemical Properties of Proteins Using Data Mining Models, PLoS ONE 8(3): e58772 10.1371/journal.pone.0058772 23505559 PMC3591381

[15] Guan P, Huang D, He M, Zhou B (2009) Lung cancer gene expression database analysis incorporating prior knowledge with support vector machine-based classification method. Journal of Experimental & Clinical Cancer Research, 28: 103 10.1186/1756-9966-28-103 19615083 PMC2719616

[16] Chen Y, Shi JX, Pan XF, Feng J, Zhao H (2013) Identification of candidate genes for lung cancer somatic mutation test kits. Genetics and molecular biology 36(3): 455–464. 10.1590/S1415-47572013000300022 24130455 PMC3795175

[17] Adetiba E, Olugbara OO (2015) Lung Cancer Prediction Using Neural Network Ensemble with Histogram of Oriented Gradient Genomic Features. The Scientific World Journal Vol. 2015(786013):1–17 10.1155/2015/786013. PMC4352926 25802891

[18] Nguyen TT, Nguyen LM Shimazu A (2007) Improving the Accuracy of Questions Classification with Machine Learning. In Proceedings of 2007 IEEE International Conference on Research, Innovation and Vision for the Future: 234–241.

[19] Rushdi A, Tuqan J (2008) The role of the symbolic-to-numerical mapping in the detection of DNA periodicities. In IEEE International Workshop on Genomic Signal Processing and Statistics, 2008 (GENSiPS): 1–4.

[20] Zhang R, Zhang CT (2014) A brief review: The z-curve theory and its application in genome analysis. Current genomics 15(2): 78 10.2174/1389202915999140328162433 24822026 PMC4009844

[21] Pruitt KD, Harrow J, Harte RA, Wallin C, Diekhans M, Maglott DR, et al (2009) The consensus coding sequence (CCDS) project: Identifying a common protein-coding gene set for the human and mouse genomes. Genome research 19(7): 1316–1323. 10.1101/gr.080531.108 19498102 PMC2704439

[22] Forbes SA, Bindal N, Bamford S, Cole C, Kok CY, Beare D, et al (2010) COSMIC: mining complete cancer genomes in the Catalogue of Somatic Mutations in Cancer. Nucleic acids research, gkq929. 10.1093/nar/gkq929 PMC3013785 20952405

[23] COSMIC (Catalogue of Somatic Mutations in Cancer). Available: http://cancer.sanger.ac.uk/cosmic/browse/tissue#sn=lung&ss=all&hn=carcinoma&sh=all&in=t&src=tissue&all_data=n. Accessed 25 June 2015.

[24] Takahashi T, Nau MM, Chiba I, Birrer MJ, Rosenberg RK, Vinocour M, et al (1989) p53: a frequent target for genetic abnormalities in lung cancer. Science 246(4929): 491–494. 2554494 10.1126/science.2554494

[25] Packenham JP, Taylor JA, White CM, Anna CH, Barrett JC, Devereux TR (1995) Homozygous deletions at chromosome 9p21 and mutation analysis of p16 and p15 in microdissected primary non-small cell lung cancers. Clinical cancer research 1(7): 687–690. 9816033

[26] Sanchez-Cespedes M, Parrella P, Esteller M, Nomoto S, Trink B, Engles JM, et al (2002) Inactivation of LKB1/STK11 is a common event in adenocarcinomas of the lung. Cancer research 62(13): 3659–3662. 12097271

[27] Rodenhuis S, Slebos RJ, Boot AJ, Evers SG, Mooi WJ, Wagenaar SS, et al (1988) Incidence and possible clinical significance of K-ras oncogene activation in adenocarcinoma of the human lung. Cancer Research 48(20): 5738–5741. 3048648

[28] Pao W, Miller V, Zakowski M, Doherty J, Politi K, Sarkaria I, et al (2004) EGF receptor gene mutations are common in lung cancers from “never smokers” and are associated with sensitivity of tumors to gefitinib and erlotinib. Proceedings of the National Academy of Sciences of the United States of America, 101(36): 13306–13311. 15329413 10.1073/pnas.0405220101 PMC516528

[29] Sasaki H, Okuda K, Kawano O, Endo K, Yukiue H, Yokoyama T, et al (2007) Nras and Kras mutation in Japanese lung cancer patients: Genotyping analysis using LightCycler. Oncology reports 18(3): 623–628. 17671710

[30] Sharma SD, Shakya K, Sharma SN (2011) Evaluation of DNA mapping schemes for exon detection. In *2011 International Conference on Computer*, *Communication and Electrical Technology (ICCCET)*: 71–74. IEEE.

[31] Nandy A, Harle M, Basak SC (2006) Mathematical descriptors of DNA sequences: development and applications. Arkivoc 9: 211–238.

[32] Alberts B, Johnson A, Lewis J, Raff M, Roberts K, Peter W, (2002) The Structure and Function of DNA, Molecular Biology of the Cell, 4th Edition, New Garland Science, New York Available: http://www.ncbi.nlm.nih.gov/books/NBK26821/. Accessed 11 August 2015.

[33] Abo-Zahhad M, Ahmed SM, Abd-Elrahman SA (2012) Genomic analysis and classification of exon and intron sequences using DNA numerical mapping techniques. International Journal of Information Technology and Computer Science (IJITCS) 4(8): 22.

[34] Voss RF (1992) Evolution of long-range fractal correlations and 1/f noise in DNA base sequences. Physical review letters 68(25): 3805 10045801 10.1103/PhysRevLett.68.3805

[35] Akhtar M, Epps J, Ambikairajah E (2007) On DNA numerical representations for period-3 based exon prediction. IEEE International Workshop on Genomic Signal Processing and Statistics, GENSIPS 2007: 1–4.

[36] Shao J, Yan X, Shao S (2013) SNR of DNA sequences mapped by general affine transformations of the indicator sequences. Journal of mathematical biology 67(2): 433–451. 10.1007/s00285-012-0564-3 22821208

[37] Cristea PD (2003) Large scale features in DNA genomic signals. Signal Processing, 83(4):, 871–888.

[38] Santo E, Dimitrova N (2007) Improvement of spectral analysis as a genomic analysis tool.In IEEE International Workshop on Genomic Signal Processing and Statistics, 2007. GENSIPS 2007: 1–4.

[39] Dimitrova N, Cheung YH, Zhang M (2006) Analysis and visualization of DNA spectrograms: open possibilities for the genome research. In Proceedings of the 14th annual ACM international conference on Multimedia:1017–1024.

[40] Adetiba E, Ibikunle FA, Daramola SA, Olajide AT (2014) Implementation of Efficient Multilayer Perceptron ANN Neurons on Field Programmable Gate Array Chip. International Journal of Engineering & Technology IJET-IJENS 14 (1):. 151–159.

[41] Thakur S, Adetiba E, Olugbara OO, Millham R (2015) Experimentation Using Short-Term Spectral Features for Secure Mobile Internet Voting Authentication. Mathematical Problems in Engineering, Volume 2015(564904): 1–21.

[42] Bo L., Whangbo T. (2014) A SIFT-Color Moments Descriptor for Object Recognition. In IT Convergence and Security (ICITCS), 2014 International Conference on (pp. 1–3). IEEE.

[43] Ojala T, Pietikäinen M, Mäenpää T (2002) Multiresolution gray-scale and rotation invariant texture classification with local binary patterns. IEEE Transactions on Pattern Analysis and Machine Intelligence 24(7): 971–987.

[44] Dalal N, Triggs B (2005) Histograms of oriented gradients for human detection. IEEE Computer Society Conference on Computer Vision and Pattern Recognition CVPR 2005 1: 886–893.

[45] Shapiro LG, Stockman GC (2001) Computer Vision New Jersey: Prentice Hall, Chap. 7: 1–14.

[46] Er MJ, Wu S., Lu J, Toh HL (2002) Face recognition with radial basis function (RBF) neural networks. IEEE Transactions Neural Networks, on, 13(3): 697–710. 10.1109/TNN.2002.1000134 18244466

[47] Gardner MW, Dorling SR (1998) Artificial neural networks (the multilayer perceptron)—a review of applications in the atmospheric sciences. Atmospheric environment, 32(14): 2627–2636.

[48] Derks EPPA, Pastor MS, Buydens LMC (1995) Robustness analysis of radial base function and multi-layered feed-forward neural network models. Chemometrics and Intelligent Laboratory Systems, 28(1): 49–60.

[49] Sanchez MS, Swierenga H, Sarabia LA, Derks E, Buydens(1996) Performance of multi layer feedforward and radial base function neural networks in classification and modelling. Chemometrics and Intelligent Laboratory Systems, 33 101–119.

[50] Kurban T, Beşdok E (2009) A comparison of RBF neural network training algorithms for inertial sensor based terrain classification. Sensors 9(8): 6312–6329. 10.3390/s90806312 22454587 PMC3312446

[51] Yu B, He X, Training Radial Basis Function Networks with Differential Evolution (2006) In Proceedings of IEEE International Conference on Granular Computing Atlanta GA, USA: 369–372.

[52] Oyang YJ, Hwang SC, Ou YY, Chen CY, Chen ZW (2005) Data classification with radial basis function networks based on a novel kernel density estimation algorithm. IEEE Trans. Neural Netw 16: 225–236. 15732402 10.1109/TNN.2004.836229

[53] Adetiba E, Ekeh JC, Matthews VO, Daramola SA, Eleanya MEU (2011) Estimating an optimal backpropagation algorithm for training an ANN with the EGFR exon 19 nucleotide sequence: an electronic diagnostic basis for non-small cell lung cancer (NSCLC). Journal of Emerging Trends in Engineering and Applied Sciences 2(1): 74–78.

[54] Zhang GP (2000) Neural networks for classification: a survey. IEEE Transactions on Systems, Man and Cybernetics Part C: Applications and Reviews 30 (4): 451–462.

[55] Lee CC, Chung PC, Tsai JR, Chang CI (1999) Robust radial basis function neural networks. Systems, Man, and Cybernetics, Part B: Cybernetics, IEEE Transactions on 29(6): 674–685. 10.1109/3477.809023 18252348

[56] Adetiba E, Ibikunle FA (2011) Ensembling of EGFR mutations’ based artificial neural networks for improved diagnosis of non-small cell lung cancer. International Journal of Computer Applications 20 (7): 39–47.

[57] Chen DS, Jain RC (1994) A robust backpropagation learning algorithm for function approximation. IEEE Trans. Neural Networks 5: 467–479. 18267813 10.1109/72.286917

[58] Cheng YH, Lin CS (1994) A learning algorithm for radial basis function network: With the capacity of adding and pruning neurons. In Proc., ICNN ‘94, 2: 797–801.

[59] Yu H, Xie T, Paszczynski S, Wilamowski BM (2011) Advantages of radial basis function networks for dynamic system design. Industrial Electronics, IEEE Transaction on 58(12): 5438–5450.

[60] Popescu MC, Balas VE, Perescu-Popescu L, Mastorakis N (2009) Multilayer perceptron and neural networks. WSEAS Transactions on Circuits and Systems 8(7): 579–588.

[61] Lu Z, Szafron D, Greiner R, Lu P, Wishart DS, Poulin B, et al (2004) Predicting subcellular localization of proteins using machine-learned classifiers. Bioinformatics 20(4): 547–556. 14990451 10.1093/bioinformatics/btg447

[62] Eisner R, Poulin B, Szafron D, Lu P, Greiner R (2005) Improving protein function prediction using the hierarchical structure of the gene ontology. In Proceedings of the 2005 IEEE Symposium on Computational Intelligence in Bioinformatics and Computational Biology, CIBCB'05: 1–10.

[63] Gebejes A, Huertas R (2013) Texture Characterization Based on Grey-Level Co-Occurrence Matrix. In Proceedings of the Conference Of Informatics And Management Sciences, ICTIC 2013:375–378.

[64] Zou KH, O’Malley AJ, Mauri L (2007) Receiver-operating characteristic analysis for evaluating diagnostic tests and predictive models. Circulation 115(5): 654–657. 17283280 10.1161/CIRCULATIONAHA.105.594929

